# Identification of pre-microRNAs by characterizing their sequence order evolution information and secondary structure graphs

**DOI:** 10.1186/s12859-018-2518-2

**Published:** 2018-12-31

**Authors:** Yuanlin Ma, Zuguo Yu, Guosheng Han, Jinyan Li, Vo Anh

**Affiliations:** 1 Key Laboratory of Intelligent Computing and Information Processing of Ministry of Education and Hunan Key Laboratory for Computation and Simulation in Science and Engineering, Xiangtan University, Hunan, 411105 China; 2 School of Electrical Engineering and Computer Science, Queensland University of Technology, GPO Box 2434, Brisbane, Q4001 Australia; 3 Advanced Analytics Institute, Faculty of Engineering & IT, University of Technology Sydney, P.O Box 123, Broadway, NSW 2007 Australia; 4 School of Mathematical Sciences, Queensland University of Technology, GPO Box 2434, Brisbane, Q4001 Australia

**Keywords:** Pre-microRNA, PSI-BLAST profiles, Hibert-Huang transform, Network, mRMR, SVM

## Abstract

**Background:**

Distinction between pre-microRNAs (precursor microRNAs) and length-similar pseudo pre-microRNAs can reveal more about the regulatory mechanism of RNA biological processes. Machine learning techniques have been widely applied to deal with this challenging problem. However, most of them mainly focus on secondary structure information of pre-microRNAs, while ignoring sequence-order information and sequence evolution information.

**Results:**

We use new features for the machine learning algorithms to improve the classification performance by characterizing both sequence order evolution information and secondary structure graphs. We developed three steps to extract these features of pre-microRNAs. We first extract features from PSI-BLAST profiles and Hilbert-Huang transforms, which contain rich sequence evolution information and sequence-order information respectively. We then obtain properties of small molecular networks of pre-microRNAs, which contain refined secondary structure information. These structural features are carefully generated so that they can depict both global and local characteristics of pre-microRNAs. In total, our feature space covers 591 features. The maximum relevance and minimum redundancy (mRMR) feature selection method is adopted before support vector machine (SVM) is applied as our classifier. The constructed classification model is named *MicroRNA −NHPred*. The performance of *MicroRNA −NHPred* is high and stable, which is better than that of those state-of-the-art methods, achieving an accuracy of up to 94.83% on same benchmark datasets.

**Conclusions:**

The high prediction accuracy achieved by our proposed method is attributed to the design of a comprehensive feature set on the sequences and secondary structures, which are capable of characterizing the sequence evolution information and sequence-order information, and global and local information of pre-microRNAs secondary structures. *MicroRNA −NHPred* is a valuable method for pre-microRNAs identification. The source codes of our method can be downloaded from https://github.com/myl446/MicroRNA-NHPred.

**Electronic supplementary material:**

The online version of this article (10.1186/s12859-018-2518-2) contains supplementary material, which is available to authorized users.

## Background

Mature microRNAs (miRNAs) are small single-stranded, non-coding RNAs (about 22 nucleotides in length), which play significant regulatory roles in various biological processes of animals, plants and viruses [1, 2]. There are two other forms of miRNAs: primary miRNAs (pri-miRNAs) and precursor microRNAs (pre-miRNAs). Mature miRNAs are cleaved from ^∼^ 90nt pre-miRNAs which are derived from the processing of a long pri-miRNA by a ribonucluease [3]. Precursor miRNAs have been widely studied at the earliest time, and many commercialized miRNA libraries take this form. With the advent of the post genome era and the development of sequencing technology, how to find all forms of miRNAs from millions of reads has become one of the challenging topics in bioinformatics. It is also difficult to experimentally identify the lowly expressed miRNAs or the miRNAs that are expressed in the specific tissues or in the developmental stage. On the other hand, as mature miRNAs are very short, the traditional feature engineering approaches [4] are usually failed to extract effective features from their sequences and structures. Therefore, current computational methods are focusing on the identification of pre-miRNAs instead of mature miRNAs.

These methods to identify pre-microRNAs can be grouped into four categories. The first category contains the earliest methods which are based on searching homologous genes [5]. The search process is a typical alignment problem of sequences and structures. The main alignment algorithms include the Smith-Waterman algorithm [5], the FASTA algorithm, and the BLAST algorithm [6–9]. However, these methods can only find highly homologous miRNAs with known miRNA sequences and require a large amount of computational resource for whole genomes. The second category contains comparative genome methods which predict miRNAs in the study of species of early stages. These methods mainly utilize the conservation characteristics of miRNAs and their precursor sequences in multiple species to search for the conserved sequences in the intergenic region. These sequences have a better secondary structure of stem ring. Based on comparative genomics, the limitation of predicting miRNAs is that the predicted miRNA candidates are highly conserved in multiple species, and these methods cannot be used to predict miRNAs which are not conserved [10–13]. At the same time, these methods are also subject to challenges of both time complexity and space complexity. The third category is based on conservation of binding sites of miRNAs which are the short sequences of miRNA binding the target mRNAs. These short sequences have conserved properties among multiple species [14–16]. The miRNAs and the target mRNAs usually have perfect complementary features in plants, while it does not match well in animals. Therefore, this category of methods is usually used in plants. The fourth category is based on machine learning methods [17–21].

Machine learning uses the information on sequences, structural and thermodynamic energy of pre-microRNAs. These methods can discover new, non-homologous pre-microRNAs. So, machine learning is the main approach for miRNA prediction and identification at present. The difficulty of the method is how to select the positive/negative samples which are able to describe sufficiently the whole sample space and how to find a better distinction between true/false pre-miRNAs. In addition, high false positive rates and computational complexity likely occur in the prediction of whole genome data. Thus, further improvement in sensitivity and specificity of the pre-miRNA classification is necessary. It is also a desirable task to explore a solution based on machine learning prediction.

By the problem of pre-microRNA identification, two major procedures are required: feature extraction and machine learning. In the past few decades, extracted features of pre-microRNAs are related to three sources: primary sequences, secondary structures and thermodynamical properties. Among them, the *k*-mer sequence composition (based on the primary sequence) is the most successful approach for the representation of pre-microRNAs [22]. Many studies have shown that most of pre-microRNAs have the properties of stem loop hair-pin structures [19]. Therefore, secondary structures can be predicted, and features derived from these structures, for instance, Xue et al. extracted 32 local structure features in triplet-SVM to predict human pre-microRNAs [19]. Energy characteristics are another kind of important features of pre-microRNAs [23, 24]. It is well studied that good features and positive / negative (real / pseudo pre-microRNA) datasets are the basis of constructing effective classification models.

In this study, we extract some novel features of pre-microRNAs for improving the current classification performance. To describe local or short-range sequence order information and evolution information of pre-microRNAs, we introduce PSI-BLAST profiles into the analysis of pre-microRNAs for the first time. And also, we introduce the Hilbert-Huang transform [25] for the first time, which is a time-frequency analysis method. Hilbert-Huang transforms are capable of capturing the local and long-range relationship between sequence bases. We obtain the topological parameters of small molecular networks constructed from the secondary structures of pre-microRNAs, which contain refined secondary structure information. These features are carefully selected so that they can depict both global and local characteristics of pre-microRNA structures. After these feature extraction, we apply support vector machine (SVM) as our classifier, and use the maximum relevance and minimum redundancy (mRMR) [26] method in the feature selection. Then, a new predictor *MicroRNA −NHPred* is constructed using the optimal feature set, which achieves an accuracy of up to 94.83% on a benchmark dataset. Our newly constructed predictor also improves the sensitivity and specificity of precursor miRNA identification.

## Methods

### Datasets

The benchmark dataset is adopted from [27–31], which consists of positive samples (true pre-microRNAs) and negative samples (pseudo pre-microRNAs). The set of positive samples is originated from the miRBase (released on 20 June, 2013) [32], composed of 1872 experimentally confirmed pre-microRNA sequences of homo sapiens. These sequences were filtered by the CD-HIT software [33], and the redundant sequences were filtered out with a threshold of 80% sequence identity. Finally, we obtained 1612 true pre-microRNA sequences as positive samples. Exactly as done by the literature works [17–19, 24], we used 8494 human pseudo pre-microRNAs. This set of negative samples collected from human protein coding regions was downloaded from [19]. These sequences are very similar to the real pre-microRNAs in the sequence length, the minimum base pair of their stem of hairpin structure and the maximum energy of secondary structure. In the same way as positive samples, we used the CD-HIT software to filter the sequences so that sequence similarity of the negative samples is kept below 80%. To overcome the sample imbalance problem [27, 28], 1612 sequences are selected randomly as negative samples from the filtered sequences.

The classification performance of our method in comparison with other methods was also tested on an independent test set. This test set comes from the latest released miRBase 22 [34] (released on March 2018) which contains 1917 pre-microRNA sequences of homo sapiens. Note that miRBase 20 (released on June 2013) contains only 1872 homo sapiens pre-microRNA sequences. The 78 new homo sapiens pre-microRNA sequences are used as the independent test set, which is named hsa dataset. We also used 410 non-coding datasets filtered out by us in Reference [18] as our negative test set (named ncRNA dataset). Meanwhile, we randomly selected 1000 human pseudo pre-microRNAs from the remaining 6882 sequences as our second negative test set (named human negative dataset).

### Feature extraction

We take three steps to extract different features of pre-microRNAs from PSI-BLAST profiles [35, 36], parameters of networks [37] and spectrum analysis based on the Hilbert-Huang transform [25].

#### PSI −BLAST profile −based features

The PSI-BLAST profile is represented as a so-called position specific score matrix (PSSM), which is acquired through aligning a query amino acid sequence to the NCBI’s nonredundant (NR) database using PSI-BLAST [35]. In this work, we apply this idea to nucleotide sequences.

First, we build a new database, which is composed of all the pre-microRNA sequences in the miRBase (http://www.mirbase.org/) and the 8494 human pseudo pre-microRNAs [19] and 754 non-coding RNAs studied in [18].

Second, we use PSI-BLAST to align a query nucleotide sequence in the dataset to the newly built database and to get the PSSM for the sequence. The PSSM is a matrix of size *L*×5, where *L* is the length of the query sequence and 5 is due to the 4 nucleotide symbols (*A*,*C*,*G*,*U*) and the symbol −. Its elements are 10× log*e* of the ratios between the observed base frequencies and the background base frequencies, and rounded down to the nearest integer.

Third, our feature extraction method also starts by transforming each element *s*_*ij*_ of the PSSM into $s_{ij}^{^{\prime }}$ using

1 $$ s_{ij}^{^{\prime}}=2^{0.1\times s_{ij}}.  $$

The resulting value $s_{ij}^{^{\prime }}$ is guaranteed to be non-negative even when *s*_*ij*_ is negative. We further apply normalization to the values $s_{ij}^{^{\prime }}$ so that each row sums to one. Let *f*_*ij*_ denote the normalized value of $s_{ij}^{^{\prime }}$. All the values *f*_*ij*_ form a matrix, which are called the frequency matrix (FM).

Fourth, to extract PSI-BLAST profile features, a so-called concensus sequence (CS) [38] is constructed from the FM as follows:

2 $$ \mu (i)=\mathrm{arg\ max}\{f_{ij}:\ 1\leq j\leq 4\},1\leq i\leq L.  $$

The *i*-th base *CS*(*i*) of the consensus sequence is set to be the *μ*(*i*)-th nucleotide in the nucleotide alphabet. It can be seen that a consensus sequence retains the most valuable evolutionary information from the PSSM.

Fifth, we compute

3 $$ \text{NCCS}(j)=\frac{n(j)}{L},1\leq j\leq 4,  $$

where *n*(*j*) is the number of the nucleotide *j* occurring in the CS. It gives 4 features corresponding to the nucleotide of the CS. Moreover, we include the entropy into our feature set, that is,

4 $$ \text{ECS}=-\sum\limits_{j=1}^{5}\text{NCCS}(j)\log_{e}\text{NCCS}(j).  $$

Another entropy-based feature is directly computed from FM to reflect the global characteristic of the PSSM:

5 $$ \text{EFM}=-\frac{1}{L}\sum\limits_{i=1}^{L}\sum\limits_{j=1}^{5}f_{ij} \log_{e}f_{ij}.  $$

Most of the extracted features of *k*-mer features shown in many articles are based on the original sequences. In this study, we extract their features from the CS of the original nucleotide sequences. Since a pre-microRNA sequence is too short (about 60bp-130bp), longer *k* are less likely to be exactly conserved among species. So, we computed *k*-mers with *k*=2,3 resulting in 80 (16+64) different features. At the same time, we calculate the content of GC from the consensus sequences.

In summary, for each query sequence, a total of 87 features are extracted from its PSI-BLAST profile. Our experimental results show that the features extracted from CS are more effective to discriminate between real pre-miRNAs and pseudo pre-miRNAs than those from the original nucleotide sequences.

#### Topological parameters of small molecular networks extracted from secondary structures

The pre-microRNA has a very significant secondary structure in the hairpin shape. There are many methods based machine-learning to identify pre-microRNAs which take advantage of the hairpin shape, so that the prediction accuracy has been greatly improved. There are more representative Triplet-SVM [19], iMiRNA-PseDpc [27], and properties based on networks [37] in these methods. In Refs. [39, 40], the authors have verified that the features based on networks have higher prediction accuracies. Meanwhile, in Ref. [37], Childs et al. further discussed the topological properties of the networks, which can reflect more essential characteristics of the pre-microRNAs. Therefore, in this work, we extract features based on networks constructed from the secondary structure, and the process is as follows:

Firstly, each nucleotide sequence of positive and negative samples is folded into a stem-loop secondary structure by RNAfold [41]. Secondly, we use a two-dimensional network (graph) to represent the RNA secondary structure, where all nucleotides are converted to nodes and all bonds between nucleotides are converted to edges. Network elements, including nodes and edges, can be defined by the network itself or parameters which may relate to limited or full knowledge of the network. According to [37], Childs et al. classified the network parameters into three types: local, local-global and global structural properties that can be used as a method in identification of RNA family. Here we use the summary statistics for the local-global properties, since they provide insight not only on the global level of the graph itself, but also on the level of its nodes and edges. Thirdly, all properties were calculated using the *igraphR* package (http://igraph.org) for complex networks. In this study, 24 network parameters are extracted to describe the stem-loop structure of pre-microRNAs based on previous works and experimental criteria [37] although a number of network parameters are available. We also choose the following features: degree, path length, shortest path, graph motifs, articulation point, modularity, graph density, coreness, closeness, centrality, bibliographic coupling, transitivity, cocitation coupling, diameter, node betweenness, edge betweenness, grith, constraint, hub score, and so on. A brief definition of all graph properties used in this study is provided in [37].

#### Extraction of sequence-order features based on the Hilbert-Huang transform

The features of the pre-microRNAs based on *k*-mers, with *k* small, they can only describe the short-range relationship between the nucleotide sequences. When *k* is larger, they can describe the long-range relationship of the nucleotide sequences, but the dimension of extracted feature vector is too large, which leads to the curse of dimensionality, and the classifier’s performance will be reduced. Since most of the previous methods extracted *k*-mer composition information from a nucleotide sequence (for pre-microRNAs, *k* generally takes the values 2, 3, 4), the sequence-order information is missing. Although Chen and Li [42] considered local sequence-order information based on Chou’s concept of pseudo amino acid composition, the overall prediction accuracy was not significantly improved. In order to depict the long range relationship and order information of the sequence, we introduce the Hilbert-Huang transform [25] based on the physical and chemical properties of the known dinucleotides.

**Fig. 1:**
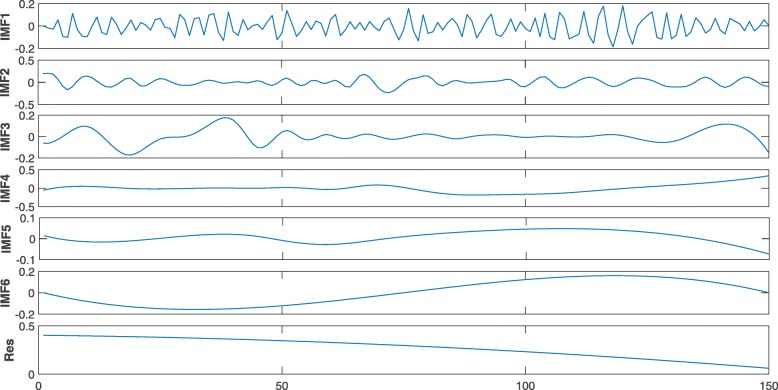
Six IMF components and the residual obtained by EMD of hydrophilicity2 time series of the pre-microRNA hsa-mir-6843

The Hilbert-Huang transformation consists of two parts: empirical mode decomposition (EMD) and Hilbert spectral analysis (HSA). The EMD method, which was originally proposed by Huang et al. [25] for the study of ocean waves, is a time-frequency analysis, and has been used by our group to simulate geomagnetic field data [43] and to predict protein subnuclear localization [44]. In EMD, the base functions, which are called intrinsic mode functions (IMFs), are obtained adaptively from the original signal. The principle and details of Hilbert spectral analysis can be found in [25, 44]. Combining the sequences of the pre-microRNAs and the physical and chemical characteristics of the dinucleotides, the feature extraction method based on the Hilbert-Huang transform is described as follows:

1. According to the physical and chemical properties of dinucleotides and the intrinsic characteristics of Hilbert-Huang transform, we selected 15 physical and chemical properties for RNAs from the database [45], including: enthalpy, enthalpy2, entropy, entropy2, free energy, free energy2, hydrophilicity, hydrophilicity2, rise, roll, shift, slide, stackingenergy, tilt, twist.
2. According to the physical and chemical properties of dinucleotides, the sequence of each pre-microRNA was converted into 15 time series by sliding a window along the sequence.
3. At first, we got the intrinsic mode functions of each time series by EMD. The EMD for a hydrophilicity2 time series of the pre-microRNA hsa-mir-6843 is shown in Fig. 1. And then we applied HSA to every intrinsic mode function to obtain the analysis signals. Finally, we obtained 32 features for each time series. The specific signal analysis process can be found in [44].

In this study, we transformed all the RNA sequences into time series according to the 15 physical and chemical properties of dinucleotides. In total, we extracted 480 Hilbert-Huang features.

### Feature selection method

After the feature extraction, some extracted features may be redundant and some may not be related to any class. There are many ways to remove redundant or useless features (in the sense that they have no significant relation to a class), such as mRMR [26], FOCUS [46], Wrapper [47], and so on. In this work, we choose the mRMR method as our feature selection method.

Let *Ω* be the whole feature space which contains all of the aforementioned 591 features in this work; each sequence is represented by a vector consisting of the values of these 591 features. We assume that *E* and *F* are two disjoint subsets of *Ω* and *Ω*=*E*∪*F*. In order to select a feature *f*_*j*_ in *E* with maximum relevance and minimum redundancy in *F*, we use the following formula:

6 $$ \max\limits_{f_{j}\in E}\left[I(f_{j},\theta)-D(f_{j},F)\right],\ j=1,2,\ldots,\sharp E,  $$

where *θ* is a vector characterizing the class of all nucleotide sequences in the sample set, *♯E* denotes the cardinality of the subset *E*, *I*(*f*_*j*_,*θ*) measures the relevance of characteristic *f*_*j*_ and class vectors *θ*, *D*(*f*_*j*_,*F*) measures the redundancy of characteristic *f*_*j*_ and the feature subset *F*. The definitions of *I*(*f*_*j*_,*θ*) and *D*(*f*_*j*_,*F*) are given in Ref. [26].

In the actual computation process, we regard *E* as a feature set to be selected, and *F* as an already selected feature set. At the beginning, *E* is the feature space, *F* is the null space, the process of the mRMR method is as follows: First, we select a feature that is most relevant to the class vector in *E*, then remove it from *E* and add it to *F*. Second, according to the mRMR function, repeat the first step. After *♯Ω* cycles, *E* is null and *F* is the entire feature set. According to the order in which the feature is added to *F*, the features in the whole feature set are reordered, and we use *S* to represent the ordered feature set:

7 $$ S=\left\{f_{i_{1}},f_{i_{2}},f_{i_{3}},\ldots,f_{i_{\sharp \Omega }}\right\}.  $$

After all features are ranked, we can determine the optimal feature components by an incremental feature selection (IFS) method [48]. For the ranked feature set *S*, we can construct the feature component sets by adding one component at a time in an ascending order as follows:

8 $$ S_{k}=\{f_{i_{1}},f_{i_{2}},f_{i_{3}},\ldots,f_{i_{k}}\}\ (1\leqq k\leqq \sharp \Omega).  $$

For each feature component set, a predictor is constructed and the accuracy is obtained by the rigorous jackknife validation. Finally, we choose the feature component set for the best jackknife validation accuracy as the optimal feature set.

### Support vector machine

A Support Vector Machine (SVM) is a class of supervised learning algorithms first introduced in [49]. It is based on statistical theory, and has a good general application. In this work, we use an SVM as a classifier to identify the real and pseudo pre-microRNAs.

Given a set of labelled training vectors (positive and negative input samples), SVM learns a linear decision boundary from both positive and negative training samples to discriminate between the unknown RNA sequences. The RNA sequence in the training set and the test set are transformed into fixed-dimension feature vectors following the process introduced above, and then the training vectors are input into SVM to construct the classifier. The SVM gives a predicted class for each RNA sequence in the test set.

The LIBSVM algorithm [50] was employed, which is a type of software for SVM classification and regression. The radial basis function (RBF) defined as

9 $$ k(\mathbf{x}_{i},\mathbf{x}_{j})=\exp\left(-\gamma (\parallel \mathbf{x}_{i}- \mathbf{x}_{j}\parallel)^{2}\right), \gamma >0  $$

is used as the kernel function *k*(**x**,**y**) in the SVM. Here, {**x**_1_,…,**x**_*n*_} is a given dataset. For a Gaussian RBF, *γ* is parametrized as $\gamma =\frac {1}{2\sigma ^{2}}$. The parameter *γ* and the soft margin parameter *C* are optimized on the benchmark dataset by adopting the grid tool provided by LIBSVM [50]. The parameters of the predictor constructed by different feature sets are shown in Table 1. More details are provided in [51].

**Table 1:** The performance of different feature sets

Method	Mcc	Accuracy	*S*_*n*_	*S*_*p*_
PSI-BLAST (*C*=512, *γ*=0.00)	0.5129	0.7564	0.7681	0.7446
HHT (*C*=2, *γ*=0.03)	0.4887	0.7440	0.7731	0.7148
Network (*C*=1024, *γ*=0.03)	0.7589	0.8785	0.9144	0.8425
PSI-BLAST+Network (*C*=1024, *γ*=0.00)	0.7707	0.8853	0.8909	0.8797
Network+HHT (*C*=1, *γ*=0.03)	0.7212	0.8802	0.8783	0.8841
PSI-BLAST+HHT+Network (*C*=4, *γ*=0.02)	0.7850	0.8973	0.9028	0.8718

### The proposed identification method

**Fig. 2:**
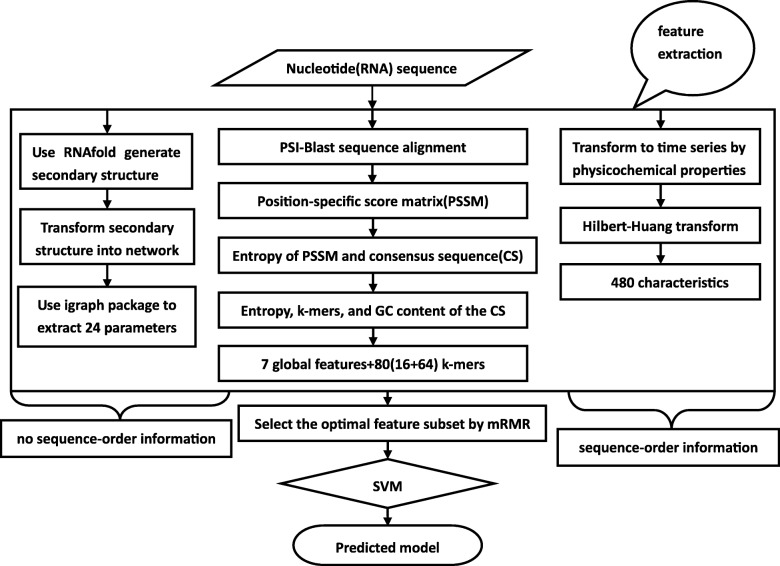
Flow chart of the identification method in this study

Figure 2 illustrates the overall architecture of our proposed method which is called *MicroRNA-NHPred*. Firstly, the query nucleotide (RNA) sequences are input into PSI-BLAST to obtain PSSM, and entropy of sequences and consensus sequences (CS) [38]. We then obtain *k*-mer composition of CS. The query nucleotide sequence is submitted to RNAfold software to generate a secondary structure.

We build a single molecule network from the secondary structure, then extract network topological parameters. Each RNA molecule is represented by the topological parameters of a single molecule network.

On the other hand, the query nucleotide sequence is converted into a time series based on the physicochemical properties of the RNA. The obtained time series are transformed and 480 characteristics are obtained. Ultimately, we get 591 features in total. These features are finally put into an SVM-based classifier for pre-microRNA classifier recognition.

### Performance evaluation

The performance of the predictor should be objectively evaluated. In statistical prediction, three cross-validation tests are often used to evaluate the prediction performance: independent dataset test, sub-sampling (or *K*-fold crossover validation) test and jackknife test. Only the jackknife test is the least arbitrary that can always yield a unique result for a given benchmark dataset [52, 53]. That is why researchers have a preference for the jackknife test for examining the quality of various machine learning based predictors such as [30, 31, 44]. Hence, in this paper we also use the jackknife test to evaluate the accuracy of the current predictor, and use independent test samples to further verify the reliability of our method. In the jackknife test, each sequence in the samples is singled out in turn as a test sample and the remaining sequences are used as training samples. Although the jackknife test consumes more computing resources, it is worthwhile to have a single output for a given set of samples.

When the cross-validation method is selected, we need to choose the performance metrics of the predictor. The identification of pre-microRNAs is a binary classification problem. For this problem, we select the following indicators to evaluate our predictor: *S*_*n*_ (sensitivity), *S*_*p*_ (specificity), Acc (overall accuracy), Mcc (Mathew correlation coefficient) [54], calculated by *S*_*n*_=*TP*/(*TP*+*FN*), *S*_*p*_=*TN*/(*TN*+*FP*), *Acc*=(*TP*+*TN*)/(*TP*+*TN*+*FP*+*FN*), and

$$Mcc=\frac{(TP\times TN)-(FP\times FN)}{\sqrt{(TP+FP)(TP+FN)(TN+FP)(TN+FN)}}. $$

In the above equations, *TP* means the true positive, *TN* the true negative, *FP* the false positive and *FN* the false negative. The sensitivity denotes correct identification of positive pre-microRNAs by avoiding false negative, while the specificity denotes correct identification of negative pre-microRNAs by avoiding false positive. The sensitivity and the specificity range between 0 and 1, the bigger the value, the better the predictor. The Mathew correlation coefficient (Mcc) ranges between -1 and 1, the overall accuracy (Acc) ranges between 0 and 1.

## Discussion and results

### Parameter selection by mRMR

We develop three steps to extract 591 features, and those features are shown in Additional file 1. Since some of these features are not essential and may not be significantly related to the classes of pre-microRNAs, we used the method in subsection “Feature selection method” to sort the features first and used the increment feature selection method to select the optimal feature set. For each feature subset, we constructed a classifier and derived its jackknife validation accuracy. Finally, we obtained the best feature subset corresponding to the best jackknife validation accuracy as the optimal feature subset. And the optimal feature set is shown in Additional file 2. We used all the feature sets to construct the predictor, whose jackknife validation accuracy turns out to be 89.73%. We used the optimal feature subset to construct a predictor with a jackknife validation accuracy of 94.83% being achieved.

At the same time, we also enumerate the top 30 features in the optimal feature set, as shown in Table 2. We can see from the Table 2 that the most relevant category of true / false pre-microRNAs is the Efm (the entropy of the frequency matrix) feature which is extracted by PSI-BLAST profiles. The average degree of node which can portray the base pairing property of RNA sequence is the second most relevant feature. In addition, we can also see that 11 features of the top 30 come from network features, 13 from HHT features, and 6 from PSI-BLAST profiles. This shows that we use three different methods to extract different levels of pre-microRNA features, which are informative and complementary.

**Table 2:** The top 30 features by feature selection

Feature	*I*(*f*_*j*_,*θ*)	Number	Feature	*I*(*f*_*j*_,*θ*)	Number
Efm	0.84881	1	CCA%	0.1727	16
A-degree	0.48748	2	hht125	0.17185	17
A-Burts	0.4461	3	hht381	0.17139	18
A-coreness	0.44058	4	hht93	0.17045	19
A-cocitation	0.31875	5	hht445	0.16982	20
A-bibliographic	0.31875	6	hht61	0.16125	21
V-coreness	0.31703	7	hht285	0.16055	22
V-coreness	0.31703	8	hht66	0.15998	23
Densith	0.31196	9	hht82	0.15998	24
Modularity	0.23591	10	(G+C)%	0.13625	25
Ecs	0.12031	11	hht94	0.15456	26
hht413	0.20155	12	CC%	0.15225	27
hht253	0.1994	13	hht157	0.15029	28
N-atriculation	0.19644	14	hht189	0.14989	29
Var-Vbetweenness	0.18213	15	GAA%	0.14786	30

### Performance of predictor on different feature sets

As shown in subsection “Feature extraction methods”, we used 3 different methods to extract 3 different feature sets. In order to study the effect of different feature sets on the performance of the predictor, we tested the single feature set and different feature combinations respectively on prediction performance, as shown in Table 1. We can see that the three feature sets have different contributions to the recognition of pre-microRNAs, of which the contribution of the network feature set is the most significant and the accuracy of the predictor is 87.85%.

We firstly introduced PSI-BLAST to the prediction of pre-microRNAs. In order to verify the performance contribution of the *k*-mers from CS, we separately extracted *k*-mers (*k*=2, 3) from the original sequence and the CS for jackknife test verification. The result of the test is shown in Table 3. The accuracy of jackknife test validation shows that the consensus sequences contain much more evolution information than the nucleotide sequences, thereby leading to more accurate pre-microRNA identification.

**Table 3:** The performance of different *k*-mers: (*k*=2,3)

Predictors	Mcc	Accuracy	*S*_*n*_	*S*_*p*_
PSI-BLAST-K-mer	0.5129	0.7205	0.7329	0.7132
*K*-mer	0.4582	0.6990	0.6780	0.7120

Secondary structure features have a variety of different representations, e.g, triplet-SVM [19], iMcRNA-PseSSC [27], network [37], and so on. To verify the effect of three secondary structure features on the problem of pre-microRNA classification, we used the jackknife test on the same benchmark dataset. As shown in Table 4, we found that the parameters of networks reflect the pre-microRNA secondary structure. So, we used the parameters of networks to depict the secondary structure of pre-microRNAs in this work.

**Table 4:** The performance of different features of secondary structure

Predictors	Mcc	Accuracy	*S*_*n*_	*S*_*p*_
Network	0.7589	0.8785	0.9144	0.8425
Triplet-SVM [19]	0.64	0.8185	0.7847	0.8520
IMcRNA-PseSSC [27]	0.72	0.8576	0.8836	0.8350

### Comparison with other methods

We compared our predictor with the best and most accurate predictors in this field, triplet-SVM [19], miPred [24], iMcRNA-EXPseSSC [27], microR-Pred (SVM) [31]. The comparison indicates that the accuracy of our predictor is higher than other predictors in the same larger and more stringent benchmark dataset using rigorous jackknife tests. As can be seen from Table 5, we have the highest prediction accuracy on Mcc, Accuracy and *S*_*n*_, and only *S*_*p*_ is lower than miPred [24] and microR-Pred (SVM) [31], but also higher than 90%.

**Table 5:** The performance of different methods on the same benchmark dataset

Predictors	Mcc	Accuracy	*S*_*n*_	*S*_*p*_
Triplet-SVM [19]	0.64	0.8185	0.7847	0.8520
MiPred [24]	0.75	0.8730	0.84	0.9060
IMcRNA-EXPseSSC [27]	0.80	0.8986	0.8993	0.8978
MicroR-Pred(SVM) [31]	0.88	0.9390	0.93	**0.9470**
*MicroRNA-NHPred* (*C*=8, *γ*=0.03)	**0.8965**	**0.9483**	**0.9490**	0.9010

The boldface represents the maximum value of each column

### Performance evaluation on an independent test set

The benchmark dataset was constructed based on miRBase released 20 (June 2013). At present, compared with miRBase released 20, the latest miRBase released 22 reports 78 new homo sapiens pre-microRNAs, which were treated as an independent test set to further evaluate the performance of the proposed *MicroRNA-NHPred*. The test results are shown in Table 6. This method trained with the benchmark dataset can correctly predict 75 testing samples in the independent dataset as true sapiens pre-microRNAs. The accuracy of the proposed method can reach 96.15%, which demonstrates the stable prediction performance of *microRNA-NHPred* for predicting sapiens pre-microRNAs.

**Table 6:** The result of different methods on an independent test set

Method	Accuracy	Pre-microRNAs which were not correctly identified
IMcRNA-EXPseSSC [27]	0.8590(67/78)	hsa-mir-8069-2, hsa-mir-1843, hsa-mir-10393, hsa-mir-10394,
		hsa-mir-10395, hsa-mir-10400, hsa-mir-10527, hsa-mir-11401,
		hsa-mir-12115, hsa-mir-12128, hsa-mir-9500;
MicroR-Pred(SVM) [31]	0.9103(71/78)	hsa-mir-10395, hsa-mir-9500, hsa-mir-8069-2, hsa-mir-12115,
		hsa-mir-10400, hsa-mir-11401, hsa-mir-12128;
*MicroRNA-NHPred*	0.9615(75/78)	hsa-mir-1843, hsa-mir-12115, hsa-mir-11401.

MicroR-Pred (SVM) [31] and iMcRNA-EXPseSSC [27], which are the most accurate predictors in this field as we know, were also tested on the same independent test set. It is worth noting that microR-Pred (SVM) [31] and iMcRNA-EXPseSSC [27] correctly identified 71 and 67 homo spaeins pre-microRNAs with an accuracy of 91.03% (71/78) and 85.90% (67/78) respectively. Our method is more accurate on these two negative independent test datasets than IMcRNA-EXPseSSC [27], but slightly less accurate than MicroR-Pred (SVM) [31], as shown in Table 7. This further confirms the reliability and validity of our method.

**Table 7:** Classification accuracy of different methods on independent test sets

Test sets	Label	Test set size	*MicroRNA-NHPred*	IMcRNA-EXPseSSC	MicroR-Pred(SVM)
hsa dataset	True	78	0.9615	0.8590	0.9103
ncRNA dataset	Pseudo	410	0.9313	0.8976	0.9390
Human negative dateset	Pseudo	1000	0.9663	0.9197	0.9726

## Conclusion

Distinction between pre-microRNAs and length-similar pseudo pre-microRNAs is a biologically important problem which can help understand more about RNA regulatory mechanisms. In this study, we have developed a new classification method called *MicroRNA-NHPred* for pre-microRNA identification. It exploits the sequence evolution information extensively from PSI-BLAST profiles, the sequence order information from Hilbert-Huang transforms and the secondary structure information from small molecule networks. A comprehensive set of 591 features is thus constructed, which depicts both global and local characteristics of sequence and secondary structure. An optimal set of 268 selected features is used by our *MicroRNA −NHPred* for the classification, and it has achieved an accuracy of up to 94.83% on same benchmark datasets.

Literature works have also used machine learning techniques to identify pre-microRNA. Our research is different in several ways, as summarized below:

1. We introduced PSI-BLAST into the analysis of pre-microRNAs. We extracted features from the consensus sequences constructed from PSSMs rather than from their respective nucleotide sequences. The former retains richer sequence evolution information. To our best knowledge, this is the first attempt to extract features from the consensus sequences.
2. We introduced the Hilbert-Huang transform into pre-microRNA identification for the first time, and used it to describe the local and long-range relationships between sequence bases.
3. We used the network parameters from the single molecule network of a pre-microRNA rather than use the triplet structure to represent the secondary structure of the pre-microRNA. These network parameters can describe more completely the local and global characteristics of RNAs. Under the same benchmark dataset, the accuracy of network parameters can reach 87.85%, while the well-known triplet-SVM reaches only 81.85%.
4. We introduced feature selection method, mRMR, into pre-microRNA identification for the first time, which yields that the accuracy of the predictor achieves 94.83% while it is 89.73% before the feature selection. The obtained results verify the significance of the feature selection.

It was observed via the rigorous cross-validation on a larger and more stringent benchmark dataset that the new predictor outperformed or was highly comparable with the best existing predictor in this area. We also performed test on an independent dataset, the results indicate that the new predictor outperforms the two best predictors for the identification of miRNAs precursor [27, 31]. This implies that the feature set obtained in this paper is highly beneficial to pre-microRNA identification. At the same time, we can conclude that hybrid features (both the primary and secondary structural features) as well as mRMR have a key role in performance improvement. If the method proposed in this paper is only used for human pre-microRNA identification, its value is limited. So, our further work is to extend it to identify pre-microRNA for cross species, and further adds some energy features to the features set.

## Additional files

Additional file 1 Describes the set of all the features extracted, which is 591. (XLSX 19.7 kb)

Additional file 2 Describes the optimal feature set, which is 268. (XLSX 15.4 kb)
